# Supplementary material for: Knowledge graph empowerment from knowledge learning to graduation requirements achievement
Source: PLoS One. 2023 Oct 12;18(10):e0292903. doi: 10.1371/journal.pone.0292903 (PMC10569641; doi:10.1371/journal.pone.0292903)
Supplement: Attachment — Submitted filename: Response to Reviewers.docx [file pone.0292903.s001.docx]

Dear Reviewers:

Thanks for your letter and comments concerning our manuscript entitled “Knowledge Graph Empowerment from Knowledge Learning to Graduation Requirements Achievement” (ID: PONE-D-23-17221). Those comments are all valuable and very helpful for revising and improving our paper, as well as the important guiding significance to our researches. We have studied comments carefully and have made correction which we hope meet with approval.

**Reviewer #1：**

*Discussing state-of-the-art knowledge graph representation learning approaches with contrastive learning would be beneficial for the paper. Contrastive learning has shown promising results in learning high-quality representations for knowledge graphs by leveraging the similarities and differences between entities. Several recent studies have proposed contrastive learning-based methods for knowledge graph representation learning, such as KGCL [1] and CKGC [2].
[1] "Knowledge graph contrastive learning for recommendation", SIGIR'2022
[2] "Cross-modal Knowledge Graph Contrastive Learning for Machine Learning Method Recommendation", ACM MM'2022
 Additionally, discussing more details about the parameter settings of compared baselines would also be beneficial for the paper. The performance of machine learning algorithms is highly dependent on the choice of hyperparameters. Therefore, providing more information about the parameter settings used for the compared baselines would enable a fairer and more accurate comparison with the proposed approach.*

**The author’s answer:**

Thank you very much for the advice you've provided us with. Your mention of discussing knowledge graph representation learning methods through contrastive learning methods is helpful for us to improve the quality of the article. Understanding that contrastive learning is an important method in knowledge reasoning, we have made additional improvements to the reasoning section of the article. Specifically, we are using large language models for knowledge reasoning: mainly by letting the knowledge graph constructed in this article, with "knowledge-course-graduation requirements" as the level of neural knowledge base, and letting the large language model learn the knowledge in the graph. We use three modules: entity extraction module, entity knowledge parsing module, and entity relationship decision module to reason about the entity relationship contained in other texts. We have marked the details in blue in the text, Specifically, lines 259-301 in the Knowledge Reasoning section. Regarding the benchmark experiments of inference performed by the Large Language Model, we have validated them in lines 342-394 in the Knowledge Graph Evaluation and Analysis section by evaluating the accuracy, relevance, and comprehensiveness of the data, and the results show that the data are of good quality. Finally, thank you again for your suggestions.

**Reviewer #2:**

*(1) The abstract needs to be rewritten by reducing some background information but including more specific results.
 (2) Why constructing a knowledge graph is necessary for this task, the research objectives, and research questions are unclear. Especially in the era of Generative AI, knowledge graph became less useful. The authors need to argue the importance and the significance of this study.
 (3) The authors did not have a good understanding on knowledge graph. There are hundreds of papers from topic conferences and journals studying the knowledge graph construction, evaluation, and applications, however, the author only cite very few references, and most of them are low quality. I believe the authors need to investigate more literature and understand the current methods for knowledge graph construction and evaluation.
 (4) The authors need to add some state-of-the-art baseline methods to demonstrate why the proposed method is more effective and useful.
 (5) The quality of the knowledge graph should be discussed and evaluated, please cite and refer to the following study:Chen, H., Cao, G., Chen, J., & Ding, J. (2019). A practical framework for evaluating the quality of knowledge graph. In Knowledge Graph and Semantic Computing: Knowledge Computing and Language Understanding: 4th China Conference, CCKS 2019, Hangzhou, China, August 24–27, 2019, Revised Selected Papers 4 (pp. 111-122). Springer Singapore
 (6) I don't think using questionnaire among students to evaluate the knowledge graph makes any sense since human this evaluation could be very subjective. I suggestion the authors to select some evaluation metrics such as correctness, relevance, comprehensive, and others for the evaluation. Also, the authors need to consider developing the downstream application such as recommendation, retrieval, or questions answering to demonstrate the usefulness of the knowledge graph, here is a paper that you can cite and refer:Nguyen, H., Chen, H., Chen, J., Kargozari, K., & Ding, J. (2023). Construction and evaluation of a domain-specific knowledge graph for knowledge discovery. Information Discovery and Delivery.
 (7) The diagrams and figures are low-quality and hard to understand. The writing of the paper is very poor*.

**The author’s answer:**

Thank you very much for the suggestions you made to us, it was very valuable to us, we have made the changes as you suggested and marked them in red in the article, for the rest of the questions you have asked, I will answer and explain one by one.

(1) We have rewritten the abstracts to reduce the amount of background information in order to make them more concise and focused on the core of the study. At the same time, we have ensured that more specific findings are included in the abstract to enable the reader to better understand the significance of our findings and discoveries. We hope that these improvements will meet your expectations and further enhance the quality of the abstracts. Thank you again for your valuable comments.

(2) The main objective of our research is to enable students to visualize the connection between knowledge points and graduation requirements, which can be easily appreciated through the structured path of knowledge graph, and can be personalized for self-directed learning based on the content of the knowledge graph. The existing generative AI, on the other hand, despite its great potential in the field of natural language processing, can be used in applications such as automatic text generation, machine translation, dialog systems, intelligent assistants, etc., which can help to improve human-computer interaction and cross-linguistic communication. However, it also has some problems, such as having randomness and easily generating answers that are not related to the question at hand; in terms of vertical domains, it may introduce noise from other domains, and so on. Therefore, considering the above, we choose to construct a knowledge graph to achieve our purpose. In addition, we have added relevant notes in the Abstract section, Lines 108-117 in the Introduction section, Lines 147-151 in the Related Work section, and Lines 425-438 in the Usage Effectiveness Analysis section to emphasize more on the purpose of our study.

(3) Thanks to your suggestion, we have conducted in-depth and extensive literature reading and research to better understand the current methods of knowledge graph construction and evaluation. At the same time, we have supplemented and improved the citations of related literature, such as literature [15]、[17]、[23]、[31]、[32], and we have marked the modified parts in red. In the order in which the literature is cited, specifically in lines 120-121, 124-127, 177, 328-332, 333-337Thanks again for your suggestions.

(4)Thanks to your suggestion, we used the current advanced large language model T5 for knowledge extraction, and objectively assessed the data quality from the perspective of comprehensiveness, relatedness and correctness, and the experimental results proved that the data quality is better, and The knowledge graph constructed using this data can help students intuitively and comprehensively understand the connections and differences between graduation requirements and each knowledge point. It enables students to engage in personalized learning through the navigation provided by the knowledge graph, allowing them to strengthen weak areas, fulfill relevant graduation requirements, and significantly enhance the attainment of graduation criteria. For specific details in lines 341-392 of the Knowledge Graph Assessment and Analysis section Once again, thank you for your suggestions.

(5) Your comments are valuable and applying them to the text will improve the quality of our research. We will cite and refer to relevant research you provide to support our knowledge graph quality assessment. We will explain this in lines 326-396 of the Knowledge Graph Assessment and Analysis section. Thank you very much for your advice.

(6) The suggestions you provided on knowledge graph evaluation methods and downstream applications were very valuable, and we made modifications based on your suggestions to improve the quality and usefulness of our study. Therefore, we drew on the references you provided to assess the objective aspects of this paper's graph, such as correctness, relevance, and comprehensiveness, in order to measure the quality of the knowledge graph and these metrics will help to more accurately assess the validity of our methodology and improve the credibility of the study, which we describe in detail in lines 329-395 in the Knowledge graph Evaluation and Analysis section. At the same time, considering that the purpose of the knowledge graph constructed in this paper is to better fulfill the graduation requirements for school students, investigating whether the knowledge graph can effectively improve the degree of achievement of graduation requirements for school students is equally important to the assessment of the usefulness of the knowledge graph, therefore, this paper adopts a combination of objective and subjective methods to comprehensively assess the quality of the knowledge graph's data and the effectiveness of its use. An assessment of the effectiveness of the use of the knowledge graph, specifically in lines 396-435.In addition, our next step will be to develop downstream applications based on your suggestions and conduct more in-depth research on this work. Thank you again for your suggestions.

(7) In response to the chart issue, we revisited all charts and redesigned them to ensure that they were clearer, better organized, and easier to understand, and used more appropriate color choices, line widths, and font sizes to improve visualization. Every effort was made to improve the manuscript in response to the writing problems, and some changes were made to the manuscript. These changes do not affect the content or framework of the paper. We have highlighted these changes in red in the revised document.We would like to express our sincere thanks for your enthusiastic work and hope that the revised paper will be approved by you.
